# Fractionated administration of irinotecan and cisplatin for treatment of lung cancer: a phase I study

**DOI:** 10.1038/sj.bjc.6690157

**Published:** 1999-02

**Authors:** H Ueoka, M Tabata, K Kiura, T Shibayama, K Gemba, Y Segawa, K Chikamori, T Yonei, S Hiraki, M Harada

**Affiliations:** Second Department of Medicine, Okayama University Medical School, 2-5-1 Shikata-cho, Okayama, 700-8558, Japan; Department of Medicine, National Shikoku Cancer Center, 13 Horinouchi, Matsuyama, 790-0007, Japan; Department of Medicine, Okayama Rosai Hospital, 1-10-25 Chikkoumidorimachi, Okayama, 702-8055, Japan; Department of Respiratory Medicine, National Okayama Hospital, 2-13-1 Minamigata, Okayama, 700-0807, Japan; Department of Medicine, Okayama Red Cross, Hospital, 65-1 Aoe, Okayama, 700-0941, Japan

**Keywords:** phase I study, irinotecan, cisplatin, small-cell lung cancer, non-small-cell lung cancer

## Abstract

A combination chemotherapy of irinotecan (CPT-11) and cisplatin (CDDP) has been reported to be active for lung cancer. In the previous trial, however, diarrhoea and leucopenia became the major obstacle for sufficient dose escalation of CPT-11 to improve the treatment outcome. We conducted a phase I study to investigate whether the fractionated administration of CDDP and CPT-11 at escalated dose was feasible and could improve the treatment outcome. Twenty-four previously untreated patients with unresectable non-small-cell lung cancer (NSCLC) or extensive disease of small-cell lung cancer (SCLC) were eligible. Both CDDP and CPT-11 were given on days 1 and 8, and repeated every 4 weeks. The dose of CDDP was fixed at 60 mg m^−2^ and given by 1-h infusion before CPT-11 administration. The starting dose of CPT-11 was 40 mg m^−2^, and the dose was escalated by an increase of 10 mg m^−2^. The maximally tolerated dose of CPT-11 was determined as 60 mg m^−2^ because grade 4 haematological or grade 3 or 4 non-haematological toxicities developed in six patients out of 11 patients evaluated. Diarrhoea became a dose-limiting toxicity. The objective response rates were 76% for NSCLC and 100% for SCLC. The recommended dose of CPT-11 and CDDP in a phase II study will be 50 mg m^−2^ and 60 mg m^−2^ respectively. © 1999 Cancer Research Campaign


British Journal of Cancer (1999) **79**(5/6), 984–990
© 1999 Cancer Research Campaign
Article no. bjoc.1998.0157

### Fractionated administration of irinotecan and cisplatin for treatment of lung cancer: a phase I study

**H Ueoka[1], M Tabata[1], K Kiura[1], T Shibayama[1], K Gemba[1], Y Segawa[2], K Chikamori[3], T Yonei[4], S Hiraki[5] and M Harada[1]**

[1]Second Department of Medicine, Okayama University Medical School, 2-5-1 Shikata-cho, Okayama 700-8558, Japan; [2]Department of Medicine, National Shikoku Cancer Center, 13 Horinouchi, Matsuyama 790-0007, Japan; [3]Department of Medicine, Okayama Rosai Hospital, 1-10-25 Chikkoumidorimachi, Okayama 702-8055, Japan; [4]Department of Respiratory Medicine, National Okayama Hospital, 2-13-1 Minamigata, Okayama 700-0807, Japan; [5]Department of Medicine, Okayama Red Cross Hospital, 65-1 Aoe, Okayama 700-0941, Japan

**Summary** A combination chemotherapy of irinotecan (CPT-11) and cisplatin (CDDP) has been reported to be active for lung cancer. In the previous trial, however, diarrhoea and leucopenia became the major obstacle for sufficient dose escalation of CPT-11 to improve the treatment outcome. We conducted a phase I study to investigate whether the fractionated administration of CDDP and CPT-11 at escalated dose was feasible and could improve the treatment outcome. Twenty-four previously untreated patients with unresectable non-small-cell lung cancer (NSCLC) or extensive disease of small-cell lung cancer (SCLC) were eligible. Both CDDP and CPT-11 were given on days 1 and 8, and repeated every 4 weeks. The dose of CDDP was fixed at 60 mg $m^{-2}$ and given by 1-h infusion before CPT-11 administration. The starting dose of CPT-11 was 40 mg $m^{-2}$, and the dose was escalated by an increase of 10 mg $m^{-2}$. The maximally tolerated dose of CPT-11 was determined as 60 mg $m^{-2}$ because grade 4 haematological or grade 3 or 4 non-haematological toxicities developed in six patients out of 11 patients evaluated. Diarrhoea became a dose-limiting toxicity. The objective response rates were 76% for NSCLC and 100% for SCLC. The recommended dose of CPT-11 and CDDP in a phase II study will be 50 mg $m^{-2}$ and 60 mg $m^{-2}$ respectively.

**Keywords:** phase I study; irinotecan; cisplatin; small-cell lung cancer; non-small-cell lung cancer

Irinotecan (CPT-11) is a semisynthetic derivative of camptothecin that exerts its cytotoxic activity by inhibiting a nuclear enzyme topoisomerase (Topo) I as a novel therapeutic target (Hsiang and Liu, 1988). CPT-11 has demonstrated a remarkable anti-tumour activity for both small-cell lung cancer (SCLC) and non-small-cell lung cancer (NSCLC) in phase II trials (Fukuoka et al, 1992; Masuda et al, 1992*a*). Cisplatin (CDDP), a recent key drug for treatment of lung cancer (Bonomi, 1996), has a different mechanism of action, and its overlapping toxicity with CPT-11 is minimal. Because CDDP was reported to show synergism with CPT-11 (Kudoh et al, 1993), this combination was considered to be evaluated. A phase I study of this combination for NSCLC, in which a fixed dose of CDDP (80 mg $m^{-2}$) given on day 1 was combined with an escalating dose of CPT-11 (30–70 mg $m^{-2}$) on days 1, 8 and 15, was reported (Masuda et al, 1992*b*, 1993). The maximally tolerated dose (MTD) and the recommended dose for a phase II study of CPT-11 were determined to be 70 mg $m^{-2}$ and 60 mg $m^{-2}$ respectively. In this study, a high response rate (54%) was achieved, but leucopenia and diarrhoea were dose-limiting toxicities and made further dose escalation of CPT-11 difficult (Masuda et al, 1992*b*, 1993). A phase II study conducted with this dose and schedule showed objective response rates of 48% for NSCLC (Nakagawa et al, 1993) and 78% for SCLC (Fujiwara et al, 1994). Then the following dose-escalation trial was conducted by combining recombinant human granulocyte colony-stimulating factor (rhG-CSF) with the original regimen. The dose of CPT-11 could be increased up to 80 mg $m^{-2}$ (a 33% increase above the original regimen). However, diarrhoea, a dose-limiting toxicity of CPT-11, prevented further dose escalation and the objective response rate remained at 50% (Masuda et al, 1994).

The present study was planned to investigate whether the fractionated administration of both CDDP and CPT-11 on days 1 and 8 could attenuate dose-limiting toxicities and improve the treatment outcome compared with the previous trial. The primary objective of this study was to determine the MTD of CPT-11 in combination with a fixed dose of CDDP. The other objectives included evaluation of the therapeutic activity and determination of the dose-limiting toxicity of this regimen.

*Received 18 February 1998*
*Revised 14 July 1998*
*Accepted 3 August 1998*

*Correspondence to:* H Ueoka

### MATERIALS AND METHODS

### Patient selection

Eligibility requirements for entry into the study were as follows: (1) histologically or cytologically proven lung cancer; (2) no prior chemotherapy, radiotherapy or surgery; (3) age of 75 years or less; (4) clinical stage of IIIA with bulky $N_2$, IIIB or IV for NSCLC, or extensive disease (ED) for SCLC; (5) performance status (PS) of 0–2 on the Eastern Cooperative Oncology Group (ECOG) scale (Oken et al, 1982); (6) the presence of measurable or evaluable disease; (7) adequate functions of the kidney (creatinine clearance ≥ 60 ml $min^{-1}$), liver (ALT, AST < twice the upper limit of

**Table 1** Patient characteristics

| No. | Age | Sex | Histology | Stage | PS | Dose of CPT-11 |
|---|---|---|---|---|---|---|
| 1 | 64 | Male | Adeno | IV | 2 | 40 |
| 2 | 57 | Female | Adeno | IV | 0 | 40 |
| 3 | 66 | Male | Adeno | IV | 1 | 40 |
| 4 | 62 | Male | Squamous | IIIB | 0 | 50 |
| 5 | 57 | Male | Squamous | IV | 0 | 50 |
| 6 | 66 | Male | Large[a] | IIIB | 1 | 50 |
| 7 | 69 | Male | Small | ED(IV) | 0 | 60 |
| 8 | 71 | Male | Adeno | IIIA | 1 | 60 |
| 9 | 67 | Male | Squamous | IIIA | 1 | 60 |
| 10 | 46 | Male | Adeno | IV | 1 | 70 |
| 11 | 38 | Male | Large | IV | 1 | 70 |
| 12 | 70 | Female | Small | ED(IIIB) | 0 | 70 |
| 13 | 47 | Female | Squamous | IV | 0 | 60 |
| 14 | 62 | Male | Adeno | IV | 1 | 60 |
| 15 | 65 | Male | Adeno | IIIB | 1 | 60 |
| 16 | 50 | Male | Small | ED(IV) | 1 | 60 |
| 17 | 49 | Male | Adenosquamous | IIIB | 0 | 60 |
| 18 | 62 | Male | Small | ED(IIIB) | 1 | 60 |
| 19 | 52 | Male | Small | ED(IIIB) | 0 | 60 |
| 20 | 65 | Male | Adeno | IV | 0 | 60 |
| 21 | 48 | Male | Adeno | IV | 0 | 50 |
| 22 | 61 | Male | Small | ED(IV) | 0 | 50 |
| 23 | 71 | Male | Squamous | IV | 0 | 50 |
| 24 | 57 | Male | Squamous | IIIB | 0 | 50 |

[a]The diagnosis was altered to thymic carcinoma at autopsy.

normal), and bone marrow (a leucocyte count ≥ 4000 $\mu l^{-1}$ and a platelet count ≥ 100 000 $\mu l^{-1}$); (8) no concomitant malignancies; and (9) a written form of informed consent.

### Evaluation

Staging procedures included complete history and physical examination, a complete blood cell count (CBC), standard blood chemistry profile, 24-h urine creatinine clearance, ECG, a chest radiograph, fibreoptic bronchoscopy, computerized tomographic (CT) scans of the chest and abdomen, magnetic resonance imaging of the brain, and radionuclide bone scan.

The CBC was repeated two or three times a week, and blood chemistry, 24-h urine creatine clearance, urinalysis, and chest radiograph were repeated at least once a week after initial evaluation. CT scans of the chest were repeated once a treatment cycle. After the completion of chemotherapy, each patient was restaged with all the tests used during the initial work-up.

### Treatment plan

Both CDDP and CPT-11 were given by 1-h intravenous infusion with an infusion pump on days 1 and 8. The dose of CDDP was fixed at 60 mg $m^{-2}$ and given with 100 ml of physiological saline. Ondansetron (4 mg) or granisetron (3 mg) was administered intravenously just before CDDP administration. CPT-11 dissolved in 300 ml of physiological saline was given after the administration of CDDP. After administration of CDDP and CPT-11, hydration consisting of 3000 ml of physiological saline was given. The starting dose of CPT-11 was 40 mg $m^{-2}$ and the dose was increased by 10 mg $m^{-2}$ for dose escalation. At least three patients were enrolled at each dose level. If all three patients developed the significant toxicity, which was defined as grade 4 haematological toxicity or grade 3 non-haematological toxicity except nausea or vomiting, the dose level was determined to be the MTD. If two of the three patients encountered the significant toxicity, as many as six patients in total were subjected to the same dose level. When the significant toxicity developed in more than half of the patients, the dose was also determined to be the MTD. Toxicity and response were evaluated according to the criteria of ECOG (Oken et al, 1982). Time to progression and overall survival were defined as the time from beginning of chemotherapy until first documentation of disease progression and to death respectively. No intrapatient dose escalation was performed. The treatment was repeated every 4 weeks up to four cycles unless the disease progression occurred. If grade 4 haematolological toxicity or grade 3 non-haematological toxicity such as diarrhoea was observed in the previous course, the dose of CPT-11 was reduced by 10 mg $m^{-2}$ in the next cycle. The dose of CDDP was reduced by 10 mg $m^{-2}$ for development of grade 4 haematological toxicity or by 30 mg $m^{-2}$ for development of grade 3 renal toxicity. Before the next course was started, leucocyte and platelet counts had to be at least 3500 $\mu l^{-1}$ or more and 100 000 $\mu l^{-1}$ or more respectively.

### Pharmacokinetics

A heparinized blood sample (5 ml) was obtained from the cubital vein opposite to the injection site at 12 points as follows: before and 15 and 30 min after the start of CPT-11 infusion, at the end of infusion, and 15 and 30 min, and 1, 2, 4, 8, 11 and 23 h after infusion. The blood was centrifuged immediately, and the plasma was stored at –20°C until analysis. Plasma levels of CPT-11 and SN-38 were

**Table 2** Dose escalation schedule

| Dose level | Dose (mg $m^{-2}$) of | | No. of patients | | No. of courses administered |
|---|---|---|---|---|---|
| | CPT-11 | CDDP | Enrolled | With toxicity[a] | |
| 1 | 40 | 60 | 3 | 0 | 8 |
| 2 | 50 | 60 | 3 | 0 | 11 |
| 3 | 60 | 60 | 3 | 1 | 11 |
| 4 | 70 | 60 | 3 | 2 | 6 |
| 5 | 60 | 60 | 8 | 5 | 18 |
| 6 | 50 | 60 | 4 | 0 | 11 |

[a] Grade 4 haematological or grade 3 or 4 non-haematological toxicity on ECOG grade except nausea and vomiting.

© Cancer Research Campaign 1999

**Table 3** Haematological toxicities

| | Dose of CPT-11 (mg m$^{-2}$) | | | |
|---|---|---|---|---|
| | **40** | **50** | **60** | **70** |
| No. of patients | 3 | 7 | 11 | 3 |
| Leucocyte count | | | | |
| Nadir (×10$^3$ μl$^{-1}$) | 1.7 (1.5–2.0) | 2.9 (1.0–3.8) | 3.0 (1.1–3.5) | 1.9 (0.2–2.2) |
| Days to nadir | 24 (20–28) | 21 (14–27) | 19 (15–24) | 26 (17–29) |
| Days to recovery | 10 (6–16) | 4 (3–17) | 5 (2–8) | 11 (10–12) |
| No. of patients with ECOG grade 3/4 toxicity | 2/0 | 2/0 | 4/0 | 1/1 |
| Platelet count | | | | |
| Nadir (×10$^3$ μl$^{-1}$) | 112 (90–249) | 86 (56–172) | 100 (50–181) | 103 (2–109) |
| Days to nadir | 24 (22–26) | 19 (18–25) | 20 (15–23) | 21 (20–26) |
| Days to recovery | 10 (7–12) | 5 (3–6) | 8 (4–13) | 6 |
| No. of patients with ECOG grade 3/4 toxicity | 0/0 | 0/0 | 1/1 | 0/1 |
| Haemoglobin level | | | | |
| Nadir (mg dl$^{-1}$) | 9.4 (9.0–9.9) | 9.3 (9.2–12.0) | 9.4 (8.5–12.4) | 9.8 (8.8–11.2) |
| Days to nadir | 30 (26–31) | 22 (21–30) | 23 (17–29) | 26 (29–26) |
| No. of patients with ECOG grade 3/4 toxicity | 0/0 | 0/0 | 1/0 | 0/0 |

Data are expressed as a median value (range).

determined by high-performance liquid chromatography (HPLC) as described previously (Kaneda et al, 1990). Pharmacokinetic parameters on each day were compared using the paired two-tailed Student's *t*-test.

### RESULTS

### Determination of MTD

Between November 1994 and August 1995, 24 patients were allocated in this study. One patient was evaluated only for toxicity because his disease was proven to be thymic carcinoma at autopsy, though this case was initially diagnosed as large-cell carcinoma of the lung. Characteristics of all patients are listed in Table 1. The median age was 62 years ranging from 38 to 71. There were 21 men and three women. Seventeen patients were diagnosed as NSCLC and six as SCLC. Dose escalation was conducted as shown in Table 2. Up to a dose level of 50 mg m$^{-2}$ of CPT-11, no patient developed the significant toxicity. At a dose level of 60 mg m$^{-2}$, one patient developed grade 3 diarrhoea. At a dose level of 70 mg m$^{-2}$, two patients developed grade 4 diarrhoea, and one of them also experienced grade 4 leucopenia and thrombocytopenia. This patient died of sepsis and subsequent multiorgan failure on day 22 of the treatment. Because this dose level was determined to be intolerable, we treated eight additional patients with CPT-11 at a dose of 60 mg m$^{-2}$. Among those, five patients developed significant toxicities, which included grade 3 or 4 diarrhoea in three patients, and grade 4 paralytic ileus, grade 3 hepatic toxicity, grade 3 skin rash and grade 4 thrombocytopenia each in one patient. Thus, six patients among a total of 11 patients developed significant toxicity when treated with 60 mg m$^{-2}$ of CPT-11. Therefore, the dose level of 60 mg m$^{-2}$ of CPT-11 was determined to be the MTD, and the recommended dose of CPT-11 for a phase II study was considered to be 50 mg m$^{-2}$. Then an additional four patients were treated at this recommended dose level of CPT-11 to confirm its safety. No severe toxicity was experienced at this dose level.

### Toxicity

Haematological toxicity was generally mild. Analysis of the first course of chemotherapy is shown in Table 3. At the first two dose levels (40 mg m$^{-2}$ and 50 mg m$^{-2}$ of CPT-11), no grade 4 haematological toxicity was experienced. Only grade 3 leucopenia was observed in two out of three (67%) and two out of seven patients (29%) at dose levels of 40 mg m$^{-2}$ and 50 mg m$^{-2}$ respectively. At the dose level of 60 mg m$^{-2}$, four patients developed grade 3 leucopenia. Of those, one patient developed grade 4 thrombocytopenia which continued for 13 days, and grade 3 thrombocytopenia and grade 3 anaemia were observed in one patient each. At the highest dose level (70 mg m$^{-2}$ of CPT-11), one patient developed grade 4 leucopenia and thrombocytopenia, and an additional patient developed grade 3 leucopenia. In most cases, the nadir of leucopenia or thrombocytopenia was observed around day 21, between day 14 and 29, with recovery of a leucocyte count to ≥ 4000 μl$^{-1}$ or a platelet count to ≥ 100 000 μl$^{-1}$ by at latest day 28.

Non-haematological toxicity is summarized in Table 4. The most prominent and dose-limiting toxicity was diarrhoea. At the first two dose levels, there was no severe diarrhoea. At dose levels of 60 mg m$^{-2}$ and 70 mg m$^{-2}$ of CPT-11, grade 3 or 4 diarrhoea was observed in 4 out of 11 (36%) and two out of three (67%) of the patients respectively. The severe diarrhoea occurred within 2 weeks (range, day 4–13) after the administration of CPT-11, which was usually controlled with loperamide hydrochloride. However, it took about 10 days (range, 8–23 days) to recover from the diarrhoea. One patient treated with 60 mg m$^{-2}$ of CPT-11 encountered grade 4 ileus, which developed at day 12 and continued for 16 days. Grade 3 skin rash occurred in one patient each at the dose levels of 60 mg m$^{-2}$ and 70 mg m$^{-2}$ of CPT-11. The skin rash was

*© Cancer Research Campaign 1999*

**Table 4** Non-haematological toxicities

| | Dose of CPT-11 (mg $m^{-2}$) | | | |
|---|---|---|---|---|
| | 40 | 50 | 60 | 70 |
| No. of patients | 3 | 7 | 11 | 3 |
| Diarrhoea | | | | |
| Grade 1 | 1 | 2 | 6 | 1 |
| 2 | 0 | 2 | 1 | 0 |
| 3 | 0 | 0 | 3 | 0 |
| 4 | 0 | 0 | 1 | 2 |
| Constipation | | | | |
| Grade 4 | 0 | 0 | 1 | 0 |
| Nausea and Vomiting | | | | |
| Grade 1 | 2 | 5 | 7 | 0 |
| 2 | 1 | 0 | 4 | 3 |
| Alopecia | | | | |
| Grade 1 | 2 | 6 | 7 | 1 |
| 2 | 0 | 0 | 4 | 1 |
| Skin rash | | | | |
| Grade 3 | 0 | 0 | 1 | 1 |
| Liver damage | | | | |
| Grade 3 | 0 | 0 | 1 | 0 |
| Peripheral neuropathy | | | | |
| Grade 1 | 0 | 0 | 0 | 1 |
| No. of patients with ECOG grade 3 or 4 toxicity | 0 | 0 | 6 | 2 |

transient and effectively treated with intravenous dexamethasone. Grade 3 hepatotoxicity occurred in one patient, which developed at day 7 and normalized until day 15. Nausea, vomiting, alopecia and peripheral neuropathy were also observed, but all of them were grade 1 or 2, transient and well tolerated. There was no evidence of severe pulmonary, cardiac or renal toxicity.

### Response

Clinical responses were evaluated in 17 patients with NSCLC and four patients with SCLC (Table 5). An objective response was observed even at the first level of CPT-11 (40 mg $m^{-2}$) and there was no clear relationship between the dose level of CPT-11 and the response. Although no complete response was achieved, partial response rates were 76% for NSCLC and 100% for SCLC. Median time to progression and median survival in four patients with SCLC were 9.34 (95% confidence interval (95% CI) 7.69–10.98) months and 16.83 (95% CI 12.55–21.12) months respectively. Those in 17 patients with NSCLC were 7.33 (95% CI 6.66–8.00) months and 10.72 (95% CI 8.45–12.99) months respectively.

### Pharmacokinetics

The pharmacokinetic parameters of CPT-11 and SN-38 on day 1 and day 8 are summarized in Table 6. Time courses of CPT-11 and SN-38 concentrations in plasma according to the dose of CPT-11 are shown in Figure 1 and Figure 2 respectively. The plasma concentrations of CPT-11 and SN-38 reached their maximum levels just before the end of CPT-11 infusion. Pharmacokinetic parameters at day 1 and day 8 compared by paired *t*-test were not significantly different. The mean beta-half lives of CPT-11 and SN-38 were 7.56 ± 0.65 h and 9.89 ± 0.95 h respectively. The $C_{max}$ and AUC of CPT-11 were increased along with the dose escalation of CPT-11 administration. However, those of SN-38 were not significantly different among different doses of CPT-11 of 40 mg $m^{-2}$, 50 mg $m^{-2}$ and 60 mg $m^{-2}$. One patient, who was treated with the highest dose (70 mg $m^{-2}$) and died of severe toxicities, showed markedly high levels of both $C_{max}$ (27.3 ng $ml^{-1}$ on day 1, 39.5 ng $ml^{-1}$ on day 8) and AUC (223.4 ng h $ml^{-1}$ on day 1, 273.0 ng h $ml^{-1}$ on day 8) of SN-38.

### Dose intensity

Dose intensity in cumulative courses are shown in Table 7. Median number of courses repeated were three and the reasons for drug discontinuation were as follows: no response (no further therapy in five patients and change to radiotherapy in four); change to high-dose chemotherapy as late intensification in three; change to adjuvant surgery in two; and life-threatening toxicity in two. Up to the second dose level, more than half of the patients completed four courses and received greater than 95% of the intended dose.

### DISCUSSION

We planned the present study to improve the treatment outcome of lung cancer by utilizing the synergistic effect between CDDP and CPT-11 maximally. So we fractionated the administration of both drugs on days 1 and 8 equally, and repeated at 4-week intervals. Objective response rates obtained with this regimen were 76% for NSCLC and 100% for SCLC. These results were considerably

**Table 5** Responses

| | Dose of CPT-11 (mg $m^{-2}$) | | | | |
|---|---|---|---|---|---|
| | 40 | 50 | 60 | 70 | Total |
| Non-small-cell lung cancer | | | | | |
| No. of evaluable patients | 3 | 5 | 7 | 2 | 17 |
| No. of PR (%) | 1 (33) | 5 (100) | 6 (86) | 1 (50) | 13 (76) |
| Small-cell lung cancer | | | | | |
| No. of evaluable patients | 0 | 1 | 3 | 0 | 4 |
| No. of PR (%) | | 1 (100) | 3 (100) | | 4 (100) |

PR, partial response.

© Cancer Research Campaign 1999 

**Table 6** Pharmacokinetic parameters of CPT-11 and SN-38

| | Dose of CPT-11 (mg m$^{-2}$) | | | |
|---|---|---|---|---|
| | 40 | 50 | 60 | 70 |
| *Day 1* | | | | |
| No. of patients | 2 | 3 | 8 | 2 |
| CPT-11 | | | | |
| $C_{max}$ (µg ml$^{-1}$) | 0.70 ± 0.09 | 0.90 ± 0.25 | 0.83 ± 0.07 | 1.11 ± 0.02 |
| $T_{max}$ (h) | 0.75 ± 0.25 | 1.13 ± 0.13 | 0.88 ± 0.08 | 1.13 ± 0.13 |
| AUC (µg · h ml$^{-1}$) | 3.03 ± 0.19 | 4.71 ± 0.16 | 4.17 ± 0.29 | 4.45 ± 0.22 |
| MRT (h) | 7.96 ± 0.52 | 7.58 ± 0.70 | 7.62 ± 0.21 | 6.99 ± 0.58 |
| SN-38 | | | | |
| $C_{max}$ (ng ml$^{-1}$) | 11.3 ± 0.2 | 12.1 ± 1.9 | 13.0 ± 1.5 | 19.7 ± 7.6 |
| $T_{max}$ (h) | 1.25 ± 0.0 | 1.38 ± 0.13 | 1.20 ± 0.1 | 2.00 ± 1.0 |
| AUC (ng · h ml$^{-1}$) | 126.7 ± 4.0 | 149.1 ± 7.3 | 121.4 ± 11.8 | 172.2 ± 51.2 |
| MRT (h) | 9.81 ± 0.14 | 9.95 ± 0.55 | 10.0 ± 0.4 | 9.42 ± 0.69 |
| *Day 8* | | | | |
| No. of patients | 2 | 1 | 4 | 2 |
| CPT-11 | | | | |
| $C_{max}$ (µg ml$^{-1}$) | 0.56 ± 0.09 | 0.67 | 0.99 ± 0.12 | 1.52 ± 0.07 |
| $T_{max}$ (h) | 1.13 ± 0.13 | 1.00 | 0.94 ± 0.16 | 1.00 ± 0.00 |
| AUC (µg · h ml$^{-1}$) | 3.17 ± 0.18 | 4.17 | 4.17 ± 0.33 | 6.14 ± 1.15 |
| MRT (h) | 8.03 ± 0.41 | 8.19 | 7.44 ± 0.16 | 6.56 ± 0.44 |
| SN-38 | | | | |
| $C_{max}$ (ng ml$^{-1}$) | 12.3 ± 1.3 | 10.0 | 13.5 ± 1.2 | 29.2 ± 10.4 |
| $T_{max}$ (h) | 1.50 ± 0.0 | 1.50 | 1.60 ± 0.2 | 2.00 ± 1.0 |
| AUC (ng · h ml$^{-1}$) | 154.4 ± 8.2 | 143.2 | 139.1 ± 16.8 | 225.5 ± 47.6 |
| MRT (h) | 9.82 ± 0.36 | 10.74 | 9.10 ± 0.6 | 9.07 ± 1.35 |

Data are expressed as means ± s.d.

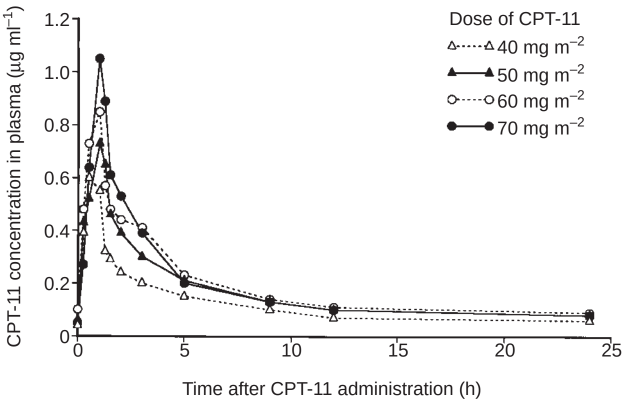

**Figure 1** Time course of CPT-11 concentration in plasma on day 1 according to the dose of CPT-11

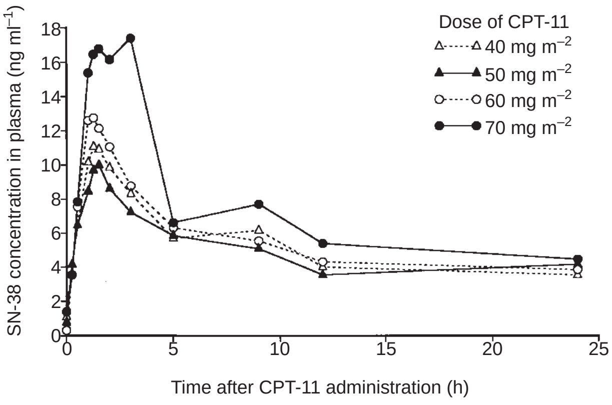

**Figure 2** Time course of SN-38 concentration in plasma on day 1 according to the dose of CPT-11

better than those of the previous reports (Masuda et al, 1992*b*, 1993, 1994; Nakagawa et al, 1993; Fujiwara et al, 1994).

As a possible explanation for these favourable results, we considered synergistic effect between CDDP and CPT-11, high dose intensity of CDDP and sequence of CDDP/CPT-11 administration. In the present study, the synergistic effect between CDDP and CPT-11 might be intensified compared with the previous trials because these drugs were simultaneously given for 2 days within one course.

We fixed the dose of CDDP at 60 mg m$^{-2}$ because we could safely give that dose of CDDP with etoposide at 200 mg m$^{-2}$ in the previous trials for SCLC (Ohnoshi et al, 1993). This regimen resulted in the increase of CDDP dose intensity (30 mg m$^{-2}$ week$^{-1}$) compared with the previous trials in which CDDP dose intensity was 20 mg m$^{-2}$ week$^{-1}$ (Masuda et al, 1992*b*, 1993, 1994; Nakagawa et al, 1993; Fujiwara et al, 1994). In contrast, the dose-intensity of CPT-11 (25 mg m$^{-2}$ week$^{-1}$) in the recommended dose of this regimen was much less than the dose intensity (45 mg m$^{-2}$ week$^{-1}$ without G-CSF or 60 mg m$^{-2}$ week$^{-1}$ with G-CSF) in the previous studies (Masuda et al, 1992*b*; 1993, 1994; Nakagawa et al, 1993; Fujiwara et al, 1994). Gralla et al (1981) and Gandara et al (1989) reported a better response rate in patients treated with

 © Cancer Research Campaign 1999

**Table 7** Dose-intensity

| Dose level | Drug | Course 1 | 2 | 3 | 4 |
|---|---|---|---|---|---|
| | | ($n$ = 3) | ($n$ = 3) | ($n$ = 2) | ($n$ = 0) |
| 1 | Cisplatin | 100 | 100 | 92 | – |
| | Irinotecan | 100 | 100 | 100 | – |
| | | ($n$ = 7) | ($n$ = 7) | ($n$ = 5) | ($n$ = 4) |
| 2 | Cisplatin | 100 | 100 | 93 | 96 |
| | Irinotecan | 100 | 97 | 96 | 95 |
| | | ($n$ = 11) | ($n$ = 8) | ($n$ = 5) | ($n$ = 3) |
| 3 | Cisplatin | 100 | 96 | 95 | 92 |
| | Irinotecan | 100 | 98 | 92 | 92 |
| | | ($n$ = 3) | ($n$ = 1) | ($n$ = 1) | ($n$ = 1) |
| 4 | Cisplatin | 100 | 86 | 86 | 86 |
| | Irinotecan | 100 | 83 | 83 | 75 |

Numbers in parentheses show number of patients evaluated. Data are expressed as administered dose/projected dose × 100.

higher doses of CDDP than in those treated with a lower dose, although Gandara's subsequent report showed that the higher dose of CDDP was harmful rather than helpful (Gandara et al, 1993). Accordingly, the increased dose intensity of CDDP may be one of the reasons for the high response rate in this study.

As for the sequence of CPT-11 and CDDP, Masuda et al (1992*b*) gave CPT-11 first on the basis of their in vitro study (Kudoh et al, 1993). However, we gave CDDP before administration of CPT-11 because this sequence was better than the inverted sequence in our in vitro study (Aoe et al, 1997). Several mechanisms are under consideration for this phenomenon. Firstly, CPT-11 may interfere with a process involved in DNA repair and enhance its cytotoxicity when given after administration of a DNA-damaging agent such as CDDP. Secondly, CDDP administration before CPT-11 may influence the excretion of SN-38. In fact, the patient who was treated with 70 mg m$^{-2}$ of CPT-11 showed higher $C_{max}$ (27.3 ng ml$^{-1}$ on day 1 and 39.5 ng ml$^{-1}$ on day 8) and equivalent AUC (172 ng h ml$^{-1}$ on day 1 and 225 ng h ml$^{-1}$ on day 8) than $C_{max}$ (13.23 ng ml$^{-1}$) and AUC (216.0 ng h ml$^{-1}$) in the previous trial using a higher dose (80 mg m$^{-2}$) of CPT-11 (Masuda et al, 1993). Similarly, in the phase I trial of combination chemotherapy with CDDP and topotecan, the sequence of CDDP before topotecan was also recommended for the subsequent trials, though this sequence induced more myelosuppression than the alternate sequence (Rowinsky et al, 1996).

Haematological toxicity in this study was generally mild and doses of CPT-11 less than 70 mg m$^{-2}$ were well tolerated. However, neither incidence nor severity of diarrhoea was improved in the present study compared with those in the previous studies, though the dose intensity of CPT-11, a main agent responsible for diarrhoea, was markedly low. These results strongly suggest the pharmacokinetic interaction between CDDP and CPT-11. A synergistic reaction between CDDP and CPT-11 in the bowel mucosa may be one of the major causes of severe diarrhoea.

Marked interpatient variability in development of toxicity is a well-known feature of CPT-11 (Fukuoka et al, 1992; Masuda et al, 1992). CPT-11 is transformed to SN-38, an active metabolite of CPT-11, by carboxylesterase, mainly in the liver, bowel mucosa and tumour tissue (Kaneda et al, 1990). Then, most of SN-38 is excreted in the bile as a glucuronate conjugate (Tsuji et al, 1991). Variability in transformation of CPT-11 to SN-38 or excretion of SN-38 may be the main cause of interpatient variability of toxicity. Treatment-related death occurred in a patient who was treated with 70 mg m$^{-2}$ of CPT-11. This patient showed a considerably high level of SN-38 in plasma. She was fully eligible for the entry criteria (ALT 41, AST 41), but her serum was positive for hepatitis C virus (HCV). Accordingly, in patients with latent hepatic dysfunction or HCV infection such as this case, SN-38 may be accumulated in plasma because of the impaired hepatic excreting ability of SN-38. In the following trials, careful examination of hepatic function may be necessary to exclude patients with latent hepatic damage. In this study, dose escalation was performed even if one of three patients experienced dose-limiting toxicities. This may have led to the very severe toxicity at the highest dose level.

In conclusion, a combination chemotherapy of CPT-11 and CDDP in this fractionating dosing schedule is feasible and highly effective for lung cancer. In seven patients who received the recommended dose for a phase II study (50 mg m$^{-2}$ of CPT-11), no patients encountered severe toxicity and all patients achieved objective responses. To confirm these encouraging results, a phase II study of CPT-11 (50 mg m$^{-2}$) and CDDP (60 mg m$^{-2}$) in the present regimen is warranted.

### REFERENCES

Aoe K, Kiura K, Ueoka H, Tabata M, Chikamori K, Kohara H and Harada M (1997) Down-regulation of topoisomerase I induced by cisplatin. *Proc Am Assoc Cancer Res* **38**: 15

Bonomi P (1996) Non-small cell lung cancer chemotherapy. In *Lung Cancer – Principles and Practice*. Pass HI, Mitchell JB, Johnson DH and Turrisi AT (eds), pp. 811–835. Lippincott-Raven: Philadelphia

Fujiwara Y, Yamakido M, Fukuoka M, Kudoh S, Furuse K, Ikegami H and Ariyoshi Y for the West Japan Lung Cancer Study Group (1994) Phase II study of irnotecan (CPT-11) and cisplatin (CDDP) in patients with small cell lung cancer (SCLC). *Am Soc Clin Oncol* **13**: 335

Fukuoka M, Niitani H, Suzuki A, Motomiya M, Hasegawa K, Nishiwaki Y, Kuriyama T, Ariyoshi Y, Negoro S, Masuda N, Nakajima S and Taguchi T for the CPT-11 Lung Cancer Study Group (1992) A phase II study of CPT-11, a new derivative of camptothecin, for previously untreated non-small-cell lung cancer. *J Clin Oncol* **10**: 16–20

Gandara DR, Wold E, Perez EA, Deisseroth AB, Doroshow J, Meyers F, McWhirter K, Hannigan J and DeGregorio MW (1989) Cisplatin dose intensity in non-small cell lung cancer: phase II results of a day 1 and day 8 high-dose regimen. *J Natl Cancer Inst* **81**: 790–794

Gandara DR, Crowley J, Livingston RB, Perez EA, Taylor CW, Weiss G, Neefe JR, Hutchins LF, Roach RW, Grunberg SM, Braun TJ, Natale RB and Balcerzak SP (1993) Evaluation of cisplatin intensity in metastatic non-small-cell lung cancer: a phase III study of the Southwest Oncology Group. *J Clin Oncol* **11**: 873–878

Gralla RJ, Casper ES, Kelsen DP, Braun DW, Dukeman ME, Martini N, Young CW and Golbey RB (1981) Cisplatin and vindesine combination chemotherapy for advanced carcinoma of the lung: a randomized trial investigating two dosage schedules. *Ann Int Med* **95**: 414–420

Hsiang Y-H and Liu LF (1988) Identification of mammalian DNA topoisomerase I as an intracellular target of the anticancer drug camptothecin. *Cancer Res* **48**: 1722–1726

Kaneda N, Nagata H, Furuta T and Yokokura T (1990) Metabolism and pharmacokinetics of the camptothecin analogue CPT-11 in the mouse. *Cancer Res* **50**: 1715–1720

Kudoh S, Takada M, Masuda N, Nakagawa K, Itoh K, Kususnoki Y, Negoro S, Matsui K, Takifuji N, Morino H and Fukuoka M (1993) Enhanced antitumor efficacy of a combination of CPT-11, a new derivative of camptothecin, and cisplatin against human lung cancer xenografts. *Jpn J Cancer Res* **84**: 203–207

Masuda N, Fukuoka M, Kusunoki Y, Matsui K, Takifuji S, Negoro S, Nishioka M, Nakagawa K and Takada M (1992*a*) CPT-11: a new derivative of camptothecin for the treatment of refractory or relapsed small-cell lung cancer. *J Clin Oncol* **10**: 1225–1229

© Cancer Research Campaign 1999

Masuda N, Fukuoka M, Takada M, Kusunoki Y, Negoro S, Matsui K, Kudoh S, Takifuji N, Nakagawa K and Kishimoto S (1992*b*) CPT-11 in combination with cisplatin for advanced non-small-cell lung cancer. *J Clin Oncol* **10**: 1775–1780

Masuda N, Fukuoka M, Kudoh S, Kusunoki Y, Matsui K, Takifuji N, Nakagawa K, Tamanoi M, Nitta T, Hirashima T, Negoro S and Takada M (1993) Phase I and pharmacological study of irinotecan in combination with cisplatin for advanced lung cancer. *Br J Cancer* **68**: 777–782

Masuda N, Fukuoka M, Kudoh S, Kusunoki Y, Matsui K, Nakagawa K, Hirashima T, Tamanoi M, Nitta T, Yano T, Negoro S, Takifuji N and Takada M (1994) Phase I study of irinotecan and cisplatin with granulocyte colony-stimulating factor support for advanced non-small-cell lung cancer. *J Clin Oncol* **12**: 90–96

Nakagawa K, Fukuoka M, Niitani H and the CPT-11 Lung Cancer Study Group (1993) Phase II study of irinotecan (CPT-11) and cisplatin in patients with advanced non-small cell lung cancer (NSCLC). *Am Soc Clin Oncol* **12**: 332

Ohnoshi T, Hiraki S, Ueoka H, Kiura K, Kamei H, Horiguchi T, Kodani T, Maeda T, Tabata M, Shibayama T, Segawa Y, Miyatake K, Takigawa N and Kimura I (1993) Pilot study of cyclophosphamide–doxorubicin–vincristine–cisplatin–etoposide hybrid chemotherapy in small cell lung cancer. *Cancer* **72**: 1597–1601

Oken MM, Davis TE, Greech RH, McFadden ET, Tormey DC, Carbone PP and Horton J (1982) Toxicity and response criteria of the Eastern Cooperative Oncology Group. *Am J Clin Oncol* **5**: 649–655

Rowinsky EK, Kaufmann SH, Baker SD, Grochow LB, Chen T-L, Peereboom D, Bowling MK, Sartorius SE, Ettinger DS, Forastiere AA and Donehower RC (1996) Sequences of topotecan and cisplatin: phase I, pharmacologic, and in vitro studies to examine sequence dependence. *J Clin Oncol* **14**: 3074–3084

Tsuji T, Kaneda N, Kado K, Yokokura T, Yoshimoto T and Tsuru D (1991) CPT-11 converting enzyme from rat serum: purification and some properties. *J Pharmacobiodyn* **14**: 341–349

 *© Cancer Research Campaign 1999*
